# Die Virushepatitiden A bis E: Prävalenz, Erregermerkmale und Pathogenese

**DOI:** 10.1007/s00103-021-03472-0

**Published:** 2021-12-21

**Authors:** Daniela Bender, Mirco Glitscher, Eberhard Hildt

**Affiliations:** Abteilung Virologie, Paul-Ehrlich-Institut – Bundesinstitut für Impfstoffe und biomedizinische Arzneimittel, Paul-Ehrlich-Straße 51–59, 63225 Langen, Deutschland

**Keywords:** Hepatitis, Hepatitisviren, Lebenszyklus, Aufbau, Pathogenese, Hepatitis, Hepatitis viruses, Life cycle, Structure, Pathogenesis

## Abstract

Bei der viralen Hepatitis handelt es sich um eine akute oder chronische Entzündung der Leber, die durch verschiedene Viren verursacht wird. Weltweit leiden derzeit ca. 325 Mio. Menschen an der chronischen Form. Jährlich versterben insgesamt ca. 1,6 Mio. an den Folgen einer viralen Hepatitis. Die Hepatitisviren werden in 5 Erregergruppen unterteilt, die mit den Buchstaben A bis E bezeichnet werden (HAV–HEV). Diese unterscheiden sich in Phylogenie, Übertragung, Epidemiologie, Wirtsspezifität, Lebenszyklus, Struktur und in speziellen Aspekten der Pathogenese.

Das strikt humanpathogene HAV, Teil der Familie *Picornaviridae*, induziert meist nur akute Hepatitiden und ist primär in Entwicklungsländern verbreitet. Das den *Hepeviridae* zugeordnete HEV beschreibt eine ähnliche Epidemiologie, ist jedoch durch sein zoonotisches Potenzial auch in Industrienationen weitverbreitet und kann zusätzlich eine chronische Erkrankung induzieren. Eine Chronifizierung tritt ebenso bei dem weltweit verbreiteten HBV (*Hepadnaviridae*) auf, dessen Satellitenvirus HDV (*Kolmioviridae)* das vorhandene kanzerogene Potenzial noch einmal erhöht. Das ebenfalls weltweit verbreitete HCV (*Flaviviridae*) birgt ein äußerst hohes Risiko der Chronifizierung und somit ebenfalls ein stark erhöhtes, kanzerogenes Potenzial.

Die Erreger der viralen Hepatitis unterscheiden sich in ihren Eigenschaften und Lebenszyklen. Eine differenzierte Betrachtung im Hinblick auf Epidemiologie, Nachweismethoden und Prävention ist daher angezeigt. Obwohl robuste Therapien, und im Falle einzelner Erreger auch Vakzine, vorhanden sind, muss die Forschung insbesondere in Hinblick auf die armutsassoziierten Erreger erheblich vorangetrieben werden.

## Abstract (en)

Viral hepatitis is characterized as an acute or chronic inflammation of the liver induced by an infection with certain viruses. At present, around 325 million humans suffer from the chronic form of the disease worldwide. Each year, about 1.6 million people die as a result of viral hepatitis. The causative agents, hepatitis viruses, are subdivided into five groups of pathogens, which are denoted with the letters A to E (HAV to HEV). These differ from each other with respect to phylogeny, transmission, epidemiology, host-specificity, life cycle, structure, and distinct aspects of pathogenesis.

The strictly human-pathogenic HAV, a member of the *Picornaviridae* family, mostly induces acute hepatitis and displays a dominant spread over the Global South. The *Hepeviridae*-affiliated HEV shows a similar epidemiology, yet spreads further into industrialized countries due to its zoonotic potential. Furthermore, HEV is defined by the capability of inducing chronic hepatitis. This course of disease is also found in a more pronounced manner for the globally prevalent HBV (*Hepadnaviridae*) and its satellite virus HDV (*Kolmioviridae*), which further increases their carcinogenic potential. Lastly, a worldwide distribution is similarly described for HCV (*Flaviviridae*), which displays a high risk of chronifications and therefore a highly increased carcinogenic potential.

The aforementioned pathogens differ with respect to their properties and life cycles. Thus, a differentiated look on epidemiology, diagnostic procedures, and disease prevention is required. Despite the presence of therapies, in some cases even a vaccine, there is an urgent need for advances in research on these aspects, especially for poverty-related pathogens.

## Hintergrund

Unter einer viralen Hepatitis versteht man eine akute oder chronische Entzündung der Leber, welche durch eine virale Infektion hervorgerufen wird. Folglich werden assoziierte Pathogene als Hepatitisviren bezeichnet, welche in die 5 Erregergruppen A bis E (HAV–HEV) unterteilt sind (Tab. 1). Diese unterscheiden sich in Phylogenie, Übertragung, Epidemiologie, Wirtsspezifität, Lebenszyklus, Struktur und in speziellen Aspekten der Pathogenese. Darüber hinaus können viele andere Erreger eine sog. Begleithepatitis auslösen.

**Table 1:** 

	Hepatitis-A-Virus	Hepatitis-B-Virus	Hepatitis-C-Virus	Hepatitis-D-Virus	Hepatitis-E-Virus
Gattung	Hepatovirus	Orthohepadnavirus	Hepacivirus	Deltavirus	Orthohepevirus
Familie	*Picornaviridae*	*Hepadnaviridae*	*Flaviviridae*	*Kolmioviridae*	*Hepeviridae*
Genom	(+)ssRNA linear	Partiell dsDNA zirkulär	(+)ssRNA linear	(−)ssRNA zirkulär	(+)ssRNA linear
Hülle	Nein, quasibehüllt	Ja	Ja	Ja	Nein, quasibehüllt
Übertragung	Fäkal-oral	Parenteral, nosokomial, perinatal	Parenteral, nosokomial, vertikal	Parenteral, Koinfektion mit HBV	Fäkal-oral, parenteral
Chronifizierung	Nein	Ja	Ja	Ja	Ja
Impfung	Ja	Ja	Nein	Nein	China
Genotypen	1–6	A–I	1–7	1‑8	1–4, 7

Derzeit leiden weltweit ca. 325 Mio. Menschen an einer chronischen viralen Hepatitis und ca. 1,6 Mio. versterben jährlich an den Folgen. Grund genug für einen knappen Überblick über diese teilweise noch immer wenig erforschten viralen Erreger. Das klinische Management der Virushepatitis bezüglich Diagnostik und Therapie wird in anderen Beiträgen dieses Themenheftes behandelt, sodass hier nicht darauf eingegangen wird.

## Hepatitis-A- und Hepatitis-E-Virus

Das Hepatitis-A-Virus (HAV) und das Hepatitis-E-Virus (HEV) sind sich in ihrer globalen Ausbreitung, den grundlegenden Zügen ihres Lebenszyklus und ihrer Pathogenese äußerst ähnlich, obgleich sie unterschiedlichen Virusfamilien angehören. Schon in der ersten Hälfte des 20. Jahrhunderts wurde eine Hepatitis des Typs A einem distinkten Pathogen zugeordnet, welches erstmals in den 1970er-Jahren per Elektronenmikroskopie visualisiert und als HAV innerhalb der Familie der *Picornaviridae* definiert wurde [1]. Auch HEV wurde nach Entdeckung in den 1980er-Jahren und erster elektronenmikroskopischer Beschreibung aufgrund seiner starken Ähnlichkeit zu HAV [2] zunächst dieser Familie zugeordnet. Nach weiterer Charakterisierung wurde HEV jedoch, aufgrund maßgeblicher Unterschiede auf genomischer Basis, der Gattung Hepevirus innerhalb der eigens angelegten Familie der *Hepeviridae* zugeordnet [3].

Global gesehen, treten sowohl HAV als auch HEV primär in Ländern mit schlechten hygienischen Standards auf. Grund hierfür ist die fäkal-orale Schmierinfektion, welche als Transmissionsroute maßgebend für die endemische Verbreitung bei unzureichenden, sanitären Verhältnissen ist [4, 5]. Jedoch stellen beide Erreger auch für Industrienationen ein Risiko dar, insbesondere durch Lebensmittelverunreinigungen (HAV) oder Zoonose (HEV; [6, 7]). Während HEV Genotyp 1 und 2 (HEV1/2) primär fäkal-oral durch kontaminiertes Trinkwasser übertragen werden, erfolgt die Verbreitung von HEV Genotyp 3 und 4 (HEV3/4) als lebensmittelbedingte Zoonose. In den Industrienationen liegt HEV3 endemisch vor und wird häufig durch unzureichend gekochtes Schweinefleisch oder den Verzehr von Wildtierfleisch wie Reh, Wildschwein oder Hase verbreitet [8]. Zusätzlich können kontaminierte Blutprodukte zur Verbreitung von HEV in Industrienationen beitragen [9]. Im Falle von HAV stellen, neben der Übertragung durch verunreinigte Lebensmittel, auch der Gebrauch von kontaminierten Spritzen beim Drogenkonsum sowie Sexualkontakte unter Männern (*Männer, die Sex mit Männern haben –* MSM) Verbreitungsmöglichkeiten dar [10].

Beide Pathogene sind somit wesentliche Verursacher von viralen, akuten Hepatitiden weltweit. HAV verursacht jährlich 1,5 Mio. symptomatische Fälle, die mit einer Sterblichkeitsrate von 0,3–1,8 % verknüpft sind und besonders ältere Menschen betreffen [11]. HEV trägt seinerseits jährlich weltweit zu 20 Mio. Fällen bei, die zu weit über 70.000 Todesfällen führen und einer Sterblichkeitsrate von 0,1–4 % bei gesunden Erwachsenen [12]. In Bezug auf die Verbreitung beträgt die Seroprävalenz von HAV in West- und Zentraleuropa zwischen 10 % und 90 % in Abhängigkeit vom Alter der untersuchten Personengruppe [13]. HEV ist im Mittel bei 24 % der west- und zentraleuropäischen Bevölkerung prävalent [14]. Im weiteren Verlauf wird auf die grundlegenden, molekularen Eigenschaften, den Lebenszyklus und die Pathogenese beider Viren eingegangen.

### Aufbau des HAV

**Figure 1:**
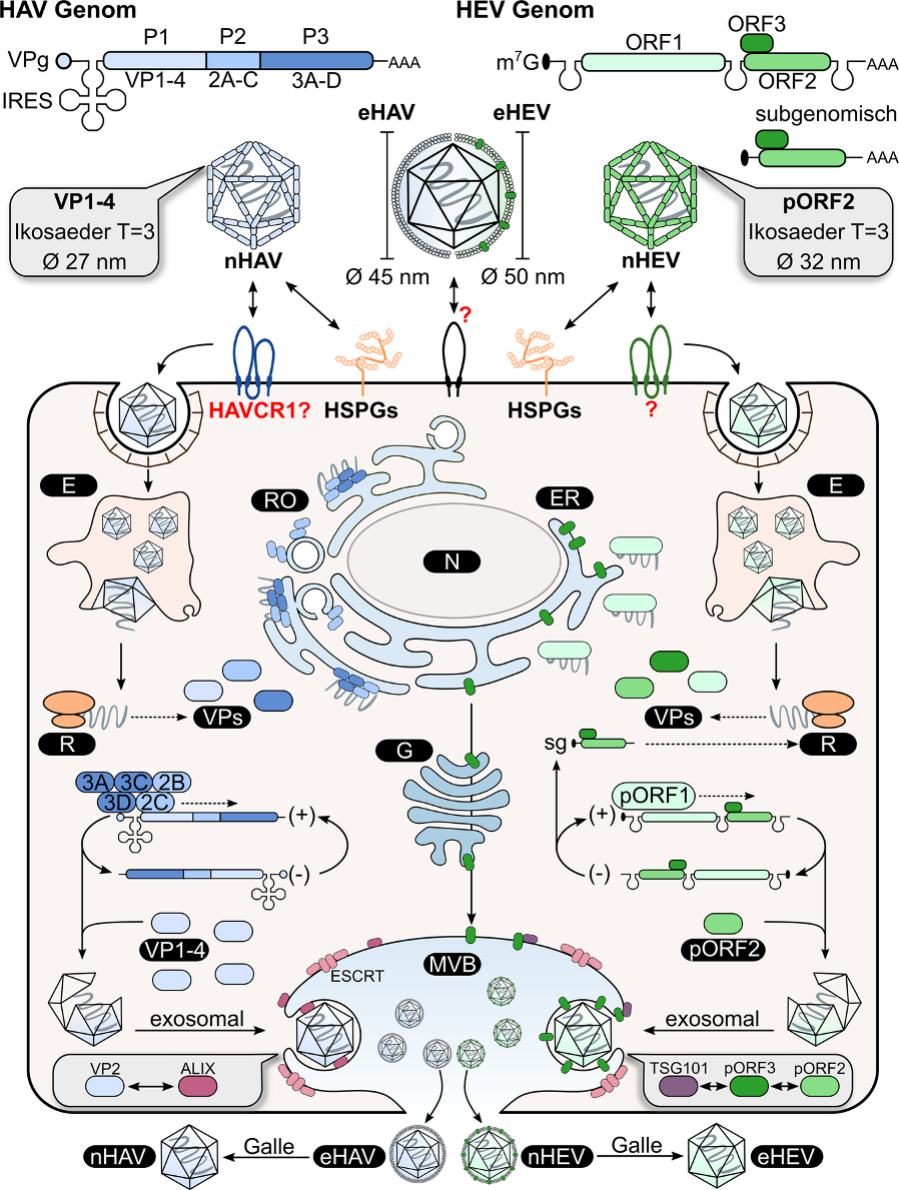

HAV ist als Teil der Familie *Picornaviridae* ein unbehülltes Virus mit ikosaedrischem Kapsid und einem positiv-gerichteten, einzelsträngigen RNA-Genom ((+)-*sense* ssRNA; Abb. 1). Letzteres umfasst ~ 7,5 Kilobasen und enthält eine Polyadenylierung innerhalb der 3′-untranslatierten Region (3′-UTR). Anders als zelluläre mRNA, ist es nicht mit einer 5′-m^7^G‑Kappe versehen, sondern durch das virale Protein VPg („viral protein genome-linked“) modifiziert. Neben der 5′- und 3′-UTR findet sich zudem ein einziger offener Leserahmen („open reading frame“ – ORF), der für das virale Polyprotein codiert, welches in die Segmente P1–3 unterteilt werden kann [15].

Segment P1 enthält Strukturproteine, die in die Untereinheiten VP1–4 eingeteilt werden. Gemeinsam vermitteln sie die Enkapsidierung des viralen Genoms, indem sie via Multimerisierung eine ikosaedrische Kapsidstruktur mit Pseudo-T = 3-Symmetrie aufbauen. Eine jede Fläche des Ikosaeders wird hierbei durch jeweils ein Molekül der Kapsidproteine gebildet. Das Kapsid umfasst schlussendlich 60 Kopien eines jeden Strukturproteins und besitzt einen Durchmesser von ca. 27 nm [16].

Aus den Segmenten P2 und P3 gehen durch proteolytische Prozessierung die Nichtstrukturproteine hervor. Diese vermitteln die membranassoziierte, genomische Replikation und werden, ähnlich wie P1, proteolytisch in die Nichtstrukturproteine 2A‑C und 3A‑D prozessiert [17].

### Lebenszyklus des HAV

Die Anlagerung und rezeptorvermittelte Endozytose des HAV wird durch das Membranprotein „HAV cellular receptor 1 protein“ (HAVCR1) eingeleitet (Abb. 1). Dieses Modell wird mittlerweile allerdings angezweifelt [18]. Die nachfolgende Internalisierung in die Hepatozyte erfolgt endosomal. Nach dem Zerfall des Nukleokapsids im Endosom gelangt das RNA-Genom in das Zytoplasma. Hierbei ist der zugrunde liegende Mechanismus noch nicht vollends geklärt. Da die virale RNA keine 5′-Kappe besitzt, wird nach Abtrennen des kovalent gebundenen VPg eine kappenunabhängige Translation durch Verwendung einer internen, ribosomalen Eintrittsstelle (IRES) innerhalb der 5′-UTR gestartet. Nach Synthese des Polyproteins werden neue Kopien des viralen Genoms über ein (−)-Strang-Intermediat durch die virale RNA-abhängige RNA-Polymerase (RdRp), welche als 3D^pol^ bezeichnet wird, synthetisiert. Die genomische Replikation ist am endoplasmatischen Retikulum (ER) angesiedelt. Hierbei induzieren die viralen Proteine 2B und 2C eine Umstrukturierung von zellulären Membranen, welche von HAV als Replikationsorganellen verwendet werden. Gesamtheitlich beinhalten diese, neben diversen Wirtsfaktoren, die viralen, membranassoziierten Proteine 2B/C und 3A‑D [19].

Im Anschluss an die genomische Replikation leitet die Multimerisierung der Proteine VP1–4 die Enkapsidierung des viralen (+)-Strang-Genoms ein. In welchem Kompartiment dies geschieht, bleibt bisher weitgehend unbekannt [15]. Obgleich HAV als nichtumhülltes Virus charakterisiert ist, nutzt es für seine Freisetzung aus infizierten Zellen keine Zelllyse, sondern membranassoziierte Prozesse. HAV wird via den ESCRT-(„endosomal-sorting-complexes-required-for-transport“-)/MVB-Weg, mittels Interaktion des viralen VP2 und des Wirtsproteins ALIX („programmed cell death 6 interacting protein“, PCD6IP) freigesetzt [15]. Als Resultat trägt HAV sowohl in Zellkultur als auch im Blut infizierter Individuen eine Membranhülle, die als quasiumhüllt bezeichnet wird und wirtseigene, jedoch keine viralen Proteine auf der Oberfläche trägt. Letztlich wird eHAV zu unbehülltem HAV (nHAV) konvertiert, indem Virionen im Gallentrakt den Gallensalzen ausgesetzt sind, welche als Detergens wirken und die Lipidhülle entfernen. Dies führt zu einer Größenänderung von ~ 45 nm (eHAV) zu ~ 27 nm (nHAV) und zur Exkretion unbehüllter Partikel im Stuhl [20].

### Aufbau des HEV

Die Familie der *Hepeviridae* ist, wie HAV, als unbehüllte Viren mit ikosaedrischem Kapsid und einem (+)-*sense* ssRNA-Genom beschrieben, welches zwischen 7,2–7,4 Kilobasen umfasst (Abb. 1). Im Unterschied zu HAV trägt HEV eine 5′-m^7^G‑Kappe und eine genomisch codierte 3′-Polyadenylierung.

In Leserichtung findet sich zuerst ORF1, welches für das virale Polyprotein pORF1 codiert, das die genomische Replikation vermittelt. Während ORF1 auf Basis der genomischen RNA translatiert wird, liegt für die Translation der Leserahmen ORF2 und ORF3 eine 2,2 Kilobasen große, subgenomische RNA vor. Diese ist folglich als bicistronische RNA zu beschreiben und wird während des Replikationszyklus gebildet. Das virale pORF2 stellt das Kapsidprotein dar. Dieses vermittelt durch Multimerisierung von 180 Monomeren des pORF2 um die virale RNA die Morphogenese des ikosaedrischen Nukleokapsids mit einer T = 3-Symmetrie und einer Größe von ca. 27–32 nm [21]. Als dritter Leserahmen codiert ORF3 für ein virales, akzessorisches Protein, welches eine Vielfalt an Aufgaben im zellulären Kontext, inklusive der viralen Freisetzung, erfüllt [22].

### Lebenszyklus des HEV

Beginnend mit der Anlagerung von Virionen an eine Zielzelle über Heparansulfat-Proteoglykane, infiziert HEV primär Hepatozyten (Abb. 1). Ein spezifischer Rezeptor ist hierbei noch nicht bekannt. Nachfolgend wird das Virus innerhalb des endosomalen Systems in die Zelle transloziert, was schließlich den Verlust der Kapsidhülle bedingt. Wie genau dieser Prozess vonstattengeht ist noch weithin unbekannt. Nach Freisetzung des viralen Genoms ins Zytoplasma wird pORF1 durch die zelluläre Translationsmaschinerie synthetisiert. Anschließend vermittelt dieses die genomische Replikation und Synthese der subgenomischen RNA über ein (−)-Strang-RNA-Intermediat. Der Ort der Replikation scheint in örtlicher Nähe zum ER zu liegen. Anschließend enkapsidiert pORF2 das Virusgenom, während pORF3 zu MVBs transloziert. Hier interagiert dieses mit dem Wirtsprotein „tumor susceptibility gene 101“ (TSG101), was wie bei HAV zu einer exosomalen Freisetzung quasiumhüllter Partikel führt. Dies hat gleichwohl zur Folge, dass eHEV (umhüllt) und nHEV (nicht umhüllt) verschiedene Mechanismen zum initialen Zelleintritt verwenden, da keine Kapsid-Rezeptor-Interaktion stattfinden kann [23]. Ebenso führt der Eintritt in den Gallentrakt durch dort vorhandene Gallensalze zur Entfernung der Lipidhülle [22]. Dies geht mit einer Änderung der Größe von ~ 50 nm (eHEV) zu ~ 30 nm (nHEV) und der Exkretion unbehüllter Virionen (nHEV) via Stuhl einher.

### Pathogenese der viralen Hepatitiden A und E

Sowohl HAV als auch HEV sind grundsätzlich als selbstlimitierende, akute Hepatitiden klassifiziert. Hierbei entwickeln lediglich 30 % der Kinder unter 6 Jahren, jedoch 70 % der Erwachsenen eine akute Hepatitis A, während eine symptomatische Hepatitis E nur in 5 % aller Transmissionen auftritt. Die Symptome sind bei beiden Erkrankungen Fieber, Übelkeit, abdominale Schmerzen und dunkler Urin bzw. heller Stuhl, welcher von Hepatomegalie und Gelbsucht bei erhöhten Transaminasewerten (AST/ALT) begleitet wird. Diese sind Konsequenz der ausgelösten Entzündungsreaktion. Ein Durchlaufen einer Infektion führt in der Regel in beiden Fällen zu einer lebenslangen Immunität [24, 25].

Neben der akuten, selbstlimitierenden Hepatitis A kann es in selteneren Fällen (ca. 10–20 %) zu einem Rezidiv kommen. Weitere Komplikationen einer HAV-Infektion sind in einem selten auftretenden (ca. 0,5–1 % der symptomatischen Fälle), akuten Leberversagen zu finden. Hiervon sind zumeist ältere Individuen betroffen, bei denen Komorbiditäten vorliegen. Dies führt zu einer Sterblichkeitsrate von ~ 10 % [24].

Ernst zu nehmende Konsequenzen im Kontext einer Infektion mit HEV sind besonders für Schwangere beschrieben. Diese entwickeln insbesondere bei Infektionen mit Genotyp 1 und 2 häufig eine fulminante Hepatitis, die zumeist in Leber- und Multiorganversagen endet und eine Sterblichkeitsrate von ca. 25 % für Schwangere bedingt. Eine zweite Risikogruppe stellen Immunsupprimierte dar, bei denen es zur Chronifizierung kommen kann [25].

Anders als bei HAV kommt es bei HEV im Kontext einer Infektion mit Genotyp 3 und 4 äußerst häufig zu einer Chronifizierung der Hepatitis, was entsprechend weitreichende, gesundheitliche Folgen nach sich zieht und nicht selten mit dem Tod des Individuums endet.

Letztlich spielen für beide Viren auch extrahepatische Manifestationen eine Rolle, wobei Niere oder Pankreas betroffen sein können sowie das zentrale Nervensystem, wo sich die Infektion als Guillain-Barré-Syndrom zeigen kann [24, 25].

## Hepatitis-B- und Hepatitis-D-Virus

Das im Jahr 1970 entdeckte Hepatitis-B-Virus (HBV) stellt weltweit eine der häufigsten Ursachen für Infektionskrankheiten dar [26]. Laut Weltgesundheitsorganisation (WHO) lag die Anzahl der chronisch mit HBV infizierten Patienten im Jahr 2019 bei weltweit 296 Mio. und führte als Folge einer Leberzirrhose oder eines Leberzellkarzinoms (HCC) zu 820.000 Todesfällen [27]. Aufgrund der Entwicklung eines sicheren und wirksamen HBV-Vakzins Anfang der 1980er-Jahre kann jedoch seit 2001 ein Rückgang der Inzidenz beobachtet werden [28]. Die Prävalenz für eine chronische HBV-Infektion liegt in Südafrika und im Westpazifik mit 5–10 % am höchsten, wohingegen die Prävalenz in Nordamerika, Westeuropa und Australien unter 1 % liegt. Die Übertragung von HBV erfolgt parenteral oder perinatal. In hochendemischen Gebieten erfolgt die Transmission hauptsächlich vertikal, wobei in Industrienationen kontaminierter Spritzengebrauch sowie häufig wechselnde Sexualkontakte erhöhte Infektionsrisiken darstellen [27].

Eine akute oder chronische HBV-Infektion kann von einer Simultan- oder Superinfektion mit dem Hepatitis-D-Virus (HDV) begleitet werden. Dieses ist ein Satellitenvirus (Virosoid), das nur in Anwesenheit von HBV repliziert. Das Virus wurde erstmals 1977 von Mario Rizzetto als HBV-assoziiertes Antigen („Delta agent“) beschrieben. Weltweit sind etwa 5 % der chronisch mit HBV infizierten Patienten mit HDV koinfiziert [29, 30]. Jedoch wird dieser Wert aufgrund einer hohen Dunkelziffer kontrovers diskutiert. In kürzlich veröffentlichten Studien wurde der Wert mit 13–14 % neu bewertet, was einer Anzahl von 50–60 Mio. (0,8 % der Weltbevölkerung) entspricht [31]. Die Prävalenz der HDV-Infektion ist geografisch ungleich verteilt. Am höchsten liegt sie in der Mittelmeerregion, in Nord- und Zentralasien, in Vietnam, auf den pazifischen Inseln, in West- und Zentralafrika sowie in Südamerika. Die Übertragung erfolgt äquivalent zu HBV. Ebenso bietet die HBV-Impfung auch einen zuverlässigen Schutz gegen HDV.

### Genomische Organisation des HBV

**Figure 2:**
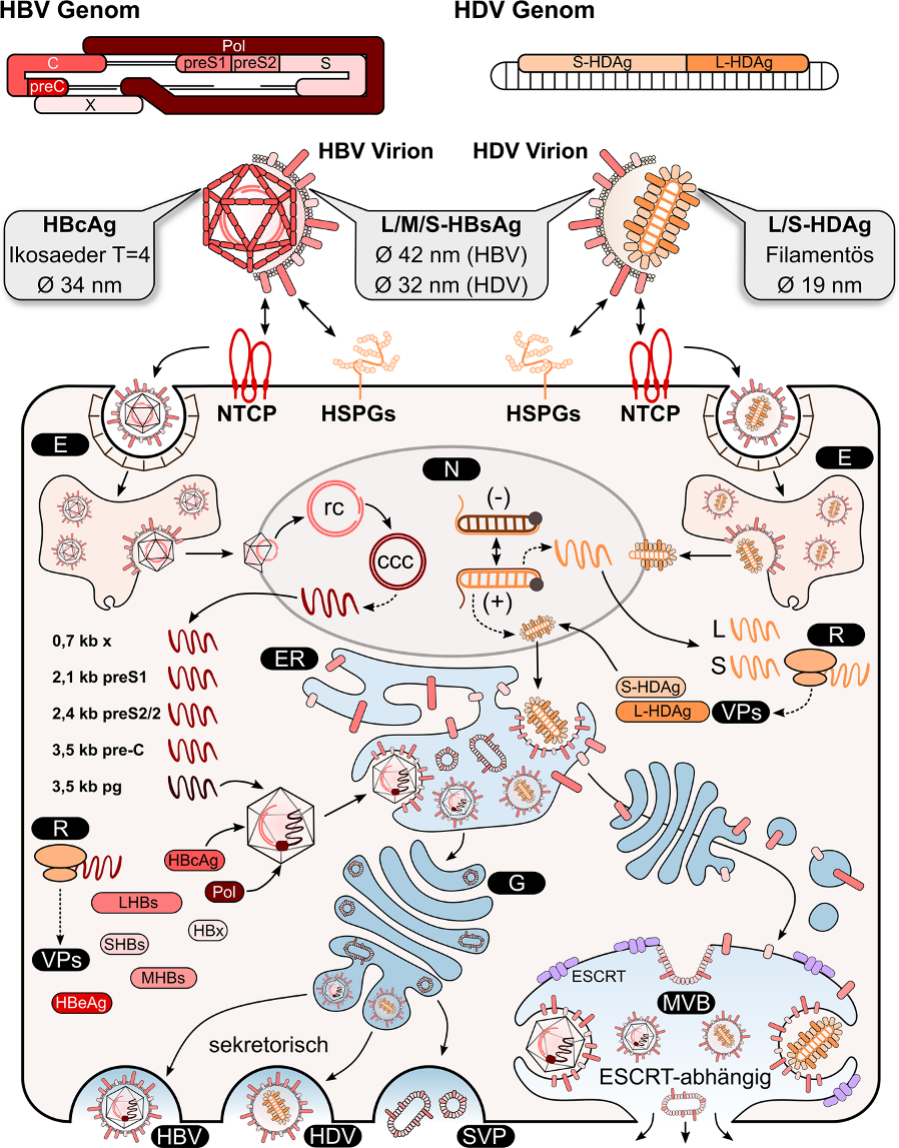

Das humane Hepatitis-B-Virus gehört zu den Orthohepadnaviren innerhalb der Familie der *Hepadnaviridae*. Das partiell doppelsträngige DNA-Genom von HBV hat eine Größe von ∼ 3,2 Kilobasen. Das Genom codiert für 4 überlappende Leserahmen (ORFs): die virale Polymerase (P), das große („large“, LHBs), das mittlere („middle“, MHBs) und das kleine („small“, SHBs) Oberflächenprotein (HBsAg), das Core-Protein (HBcAg) und seine sekretorische Variante Pre-Core (HBeAg) sowie das regulatorische X‑Protein (HBx; [32]; Abb. 2). Die HBV-Oberflächenproteine werden von einem einzigen ORF codiert, der durch 3 *In-Frame*-Startcodons in die PreS1-, PreS2- und S‑Domänen unterteilt wird. Hierbei umfasst LHBs den gesamten ORF, MHBs die PreS2- und S‑Domäne und SHBs lediglich S, welches die ER-Verankerung vermittelt [32–34].

Das Core-Protein (HBcAg) bildet die Untereinheit des HBV-Nukleokapsids. Core-Dimere bilden entweder ∼ 30 nm große Partikel, bestehend aus 90 HBcAg-Dimeren (T = 3-Symmetrie), oder Partikel einer Größe von 34 nm, bestehend aus 120 HBcAg-Dimeren (T = 4-Symmetrie). Letztere werden in 95 % der Infizierten gefunden.

Die infektiösen Partikel, auch als „Dane-Partikel“ bezeichnet, sind ∼ 42 nm groß, enthalten das Nukleokapsid und tragen die in einer Lipidhülle eingebetteten Oberflächenproteine. Neben den Dane-Partikeln werden auch subvirale, nichtinfektiöse Partikel (SVPs) freigesetzt, die nur aus der Lipidhülle und den viralen Oberflächenproteinen bestehen. Die sogenannten Sphären weisen einen Durchmesser von 22 nm auf und bestehen überwiegend aus SHBs, während die Filamente einen höheren Anteil an LHBs aufweisen. Die SVPs dienen vermutlich dem Abfangen spezifischer HBsAg-Antikörper [35].

### Lebenszyklus des HBV

HBV infiziert mit hoher Gewebs- und Speziesspezifität humane Hepatozyten. Nach unspezifischer Anlagerung an Heparansulfat-Proteoglykane (HSPGs) erfolgt die Bindung des Virus an den spezifischen Rezeptor, „Natrium-taurocholat cotransporting polypeptid“ (NTCP; Abb. 2). Ob weitere (Co‑)Rezeptoren an der Bindung und Internalisierung des Virus beteiligt sind, ist noch unklar [33]. Die Internalisierung erfolgt über clathrin-abhängige Endozytose. Nach pH-abhängiger Freisetzung der Nukleokapside aus den Endosomen werden diese gerichtet in den Zellkern transportiert, wo die rcDNA („relaxed circular DNA“) in die episomale, kovalent geschlossene, zirkuläre DNA („covalently closed circular DNA“, cccDNA) umgewandelt wird. Diese dient als Matrize für die Synthese prägenomischer (pg)RNA und subgenomischer mRNAs. Unter Kontrolle der 4 Promotoren Core, X, PreS1, PreS2 und zweier *Enhancer*-Elementen (EnhI, EnhII) entstehen die 4 entsprechenden HBV-Transkripte [32, 33, 36, 37]. Die pgRNA wird zusammen mit der HBV-Polymerase (P) in die neu gebildeten HBV-Kapside verpackt, wo sie von der viralen Polymerase (P) in rcDNA-Genome revers transkribiert wird [38, 39]. Die reifen Nukleokapside werden entweder zurück in den Zellkern transportiert, wo sie zunächst für die Vergrößerung des cccDNA-Pools sorgen, oder sie werden als unbehüllte („naked capsids“) oder reife Virionen aus der Zelle freigesetzt. Für Letzteres werden die Nukleokapside von ER-residenten Oberflächenproteinen ummantelt. Hierfür ist u. a. die LHBs-Membrantopologie mit der in Richtung Zytosol zeigenden PreS1-PreS2-Domäne durch Interaktion mit dem Nukleokapsid relevant. Die reifen Viruspartikel werden entgegen früheren Vermutungen schließlich ESCRT-(„endosomal-sorting-complex-required-for-transport“-)abhängig über den ESCRT-/*Multivesicular-Bodies-*(MVBs‑)Transportweg freigesetzt. Essenziell hierfür ist die Interaktion von LHBs mit α‑Taxilin, das als Adapter für die ESCRT-Maschinerie dient. Die Freisetzung der subviralen Filamente, die einen hohen Gehalt an LHBs aufweisen, erfolgt ebenfalls ESCRT-/MVB-abhängig, wohingegen die subviralen Sphären über den klassischen sekretorischen Weg freigesetzt werden [33, 37].

### Genomische Organisation des HDV

HDV ist einer der bisher 8 Vertreter der Gattung *Deltavirus*, in der Familie *Kolmioviridae*. Das (−)-*sense* partiell zirkuläre RNA-Genom besitzt eine Größe von 1700 Nukleotiden. Das Genom codiert für ein einzelnes Protein, das Delta-Antigen (HDAg), das in 2 unterschiedlichen Formen von 24 kDa (kleines Delta-Antigen, S‑HDAg) und 27 kDa (großes Delta-Antigen, L‑HDAg) vorliegen kann ([30, 31]; Abb. 2).

Die sphärischen HDV-Partikel, mit einem Durchmesser von 36 nm, bestehen aus dem HDV-Genom, das zusammen mit dem HDAg einen Ribonukleoproteinkomplex (RNP) bildet. Während des Lebenszyklus erhalten die Partikel die vom ER abstammende Lipidhülle, in die die Oberflächenproteine des HBV (LHBs, MHBs, SHBs) eingelagert sind. Aufgrund dessen ist der gesamte HDV-Lebenszyklus von der HBV-Infektion und der Synthese der HBV-Oberflächenproteine abhängig [30, 31].

### Lebenszyklus des HDV

Wie HBV infiziert auch HDV mit hoher Wirtsspezifität humane Hepatozyten. Die Bindung und Internalisierung der Viruspartikel erfolgen analog zu HBV, wobei der Prozess bisher nicht vollständig verstanden ist. Nach clathrin-abhängiger Endozytose wird der RNP in das Zytoplasma der Hepatozyten freigesetzt. Mittels der Kernlokalisationssequenzen des HDAg (L-HDAg, S‑HDAg) erfolgt der Import der RNP in den Zellkern. Die anschließende HDV-Replikation erfolgt HBV-unabhängig. Während der Replikation findet man in der Zelle das HDV-Genom, die antigenomische RNA sowie die mRNA für das L‑HDAg. Die 3 RNAs werden vermutlich über zelluläre Mechanismen synthetisiert [30]. Die Replikation erfolgt über einen sogenannten Rolling-Circle-Mechanismus, wobei zunächst lineare Antigenome entstehen, die durch das auf dem HDV-Genom befindliche Ribozym prozessiert und anschließend ligiert werden. Die Antigenome dienen als Template für die *De-novo-*Synthese von HDV-Genomen über einen ähnlichen Rolling-Circle-Mechanismus. Nach erfolgreicher Farnesylierung des L‑HDAg im Zytoplasma erfolgt der Transport in den Zellkern, wo das L‑HDAg die Verpackung der neu synthetisierten HDV-Genome in neue Viruspartikel einleitet. Die RNPs werden über das Kernexportsignal im L‑HDAg in das Zytoplasma transloziert und zum ER transportiert, wo die Interaktion mit HBV-Oberflächenproteinen stattfindet. Anschließend verlassen die ca. 36–45 nm großen Viruspartikel die Zelle über einen noch unbekannten Mechanismus [31].

### Pathogenese einer viralen Hepatitis B und Hepatitis D

Eine Infektion mit dem Hepatitis-B-Virus kann zu einer akuten Hepatitis (mit oder ohne fulminanten Verlauf), zu einer chronischen Hepatitis, einer Leberzirrhose und zu einem hepatozellulären Karzinom (HCC) führen. Bei etwa 2 Dritteln der akut Infizierten verläuft die Erkrankung asymptomatisch oder mit milden, grippeähnlichen Symptomen. Nur ein Drittel der Infizierten entwickelt eine ikterische Hepatitis mit schwerwiegenden Symptomen wie starker Übelkeit und Erbrechen sowie Schmerzen im Oberbauch. Bei etwa einem Prozent aller Infizierten kann sich eine lebensbedrohliche, fulminante Hepatitis mit akutem Leberversagen manifestieren. Bei über 90 % der Infizierten heilt die Hepatitis-B-Infektion jedoch vollständig aus und vermittelt eine lebenslange Immunität [28, 31, 32].

Abhängig vom Alter zum Zeitpunkt der Infektion kann sich eine chronische Hepatitis entwickeln (bei perinataler Infektion chronifiziert die Infektion bei 80–90 %, bei immunkompetenten Erwachsenen jedoch nur bei wenigen Prozent). Sie ist eine der Hauptursachen für die Entwicklung einer Leberfibrose, einer Leberzirrhose und eines HCC [37]. Die Immunpathogenese der chronischen Hepatitis-B-Infektion ist komplex und nicht vollständig verstanden. Die Unfähigkeit des adaptiven Immunsystems, die Infektion vollständig zu eliminieren, führt zu einer sich wiederholenden Zerstörung der Hepatozyten im Wechsel mit einer Regeneration. Über Jahre führt dies zu einem fibrotischen Prozess und somit zur Entstehung einer Leberzirrhose oder eines HCC [37, 40, 41]. Die Pathogenese der viralen Hepatitis B wird ausführlicher im Beitrag von Glitscher et al. in diesem Themenheft dargestellt.

Die HDV-Infektion kann entweder als Simultaninfektion einer akuten HBV-Infektion oder als Superinfektion einer chronischen HBV-Infektion vorkommen. In beiden Fällen ist das Risiko einer fulminanten Hepatitis stark erhöht. Die HDV-Simultaninfektion kann in 95 % der Fälle eliminiert werden, wohingegen die Superinfektion eines HBV-Trägers mit HDV bei 80 % der Infizierten zu einem chronischen Verlauf führt. Dies erhöht die Wahrscheinlichkeit, eine Leberfibrose oder Leberzirrhose zu entwickeln, um den Faktor 10 und verdreifacht das Risiko, an einem HCC zu erkranken [31].

## Das Hepatitis-C-Virus

Das Hepatitis-C-Virus wurde erstmals 1988 im Serum eines Posttransfusionspatienten mit einer Non-A-Non-B-Hepatitis (NANBH) diagnostiziert [42]. Nach Schätzungen der WHO sind derzeit 58 Mio. Menschen mit dem Hepatitis C Virus infiziert, 290.000 Menschen sterben jährlich an den Folgen einer Infektion [43, 44]. Die Infektion mit dem Hepatitis-C-Virus ist nicht nur ein Problem der Entwicklungsländer, sondern stellt auch die Industrienationen vor eine große Herausforderung. Laut WHO liegt die Prävalenz in Ländern der östlichen Mittelmeerregion (WHO-Region EMRO; [45]) am höchsten (2,3 %), gefolgt von Europa und den USA (1,5 %; [43, 44]).

Die Übertragung erfolgt hauptsächlich parenteral, wobei nosokomiale Übertragungen sowie solche durch kontaminierte Spritzen beim Drogenkonsum die Hauptursachen für eine HCV-Infektion darstellen [44]. Bislang ist keine Schutzimpfung verfügbar. Jedoch ist mit der Entwicklung und Zulassung neuer direkt antiviral wirksamer Medikamente (DAAs; „direct acting antivirals“) eine deutliche Verbesserung der Therapie gegeben [46].

### Genomische Organisation des HCV

**Figure 3:**
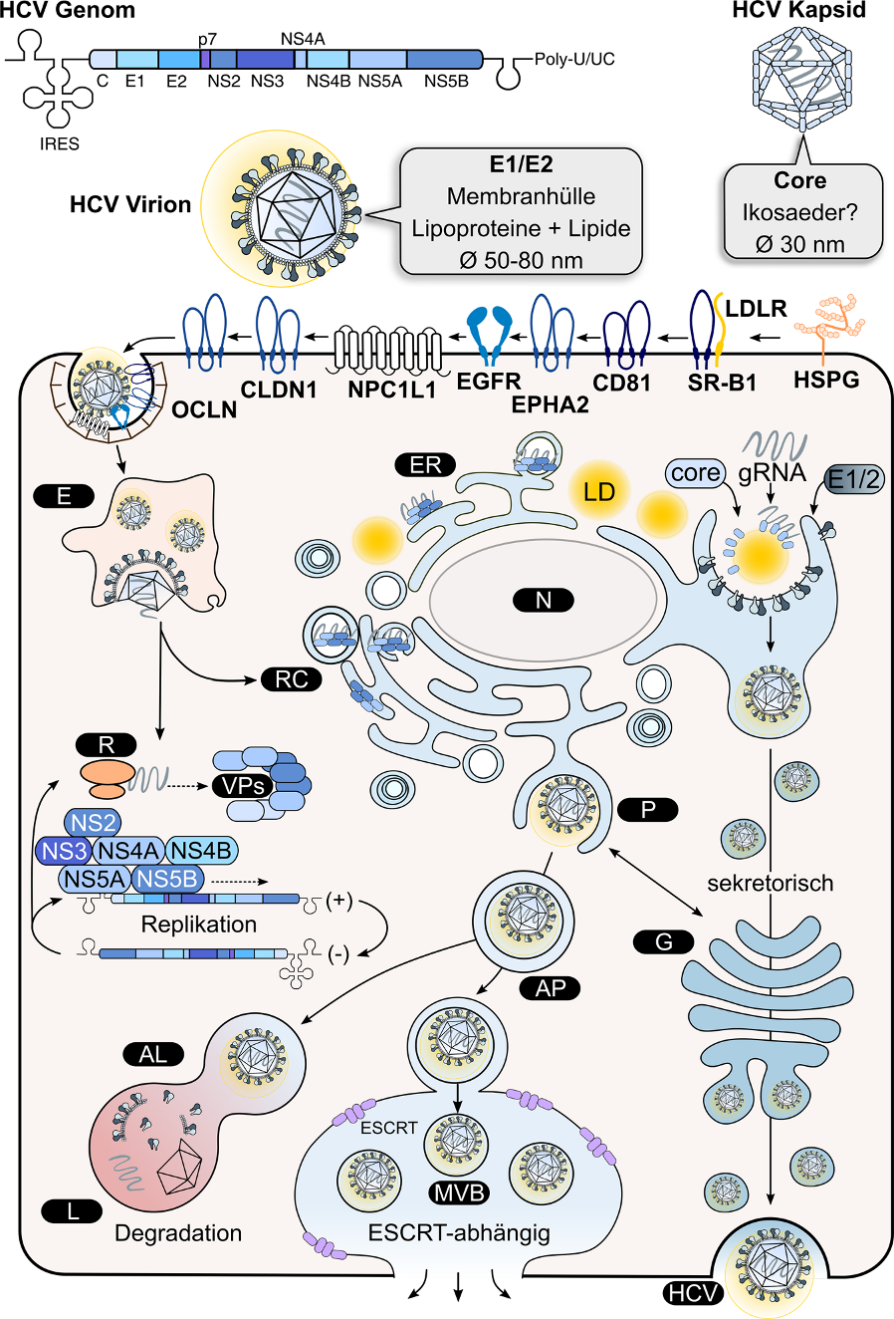

HCV ist ein RNA-Virus, das zu den Hepaciviren innerhalb der *Flavivirus-*Familie gehört. Die (+)-*sense* ssRNA besitzt eine Größe von 9600 Nukleotiden und wird von 5′- und 3′-UTR (*untranslatierten Regionen*) flankiert (Abb. 3). Die IRES-abhängige Translation resultiert in der Synthese eines Vorläuferpolyproteins, das co- und/oder posttranslational von viralen und zellulären Proteasen in die 10 reifen viralen Proteine prozessiert wird. Die N‑terminalen Strukturproteine (Core, E1, E2) sind am Aufbau der Viruspartikel beteiligt, wohingegen die Nichtstruktur-(NS-)Proteine (p7, NS2, NS3, NS4A, NS4B, NS5A, NS5B) die Genomreplikation vermitteln und regulatorische Aufgaben im HCV-Lebenszyklus sowie innerhalb zellulärer Prozesse übernehmen [47].

Die HCV-Virionen besitzen eine sphärische Struktur und haben einen Durchmesser von 50 nm. Die Partikel sind von einer Lipidhülle umgeben, in die die viralen Glykoproteine E1 und E2 eingelagert sind. Sie umgibt das Nukleokapsid, das aus homooligomerisierten Core-Proteinen besteht und mit der viralen RNA assoziiert ist [48]. Die HCV-Partikel sind stark mit Lipiden assoziiert, weshalb sie auch als Lipoviropartikel (LVP) bezeichnet werden. Folglich variiert die Dichte der LVP zwischen 1,03–1,25 g/cm^3^, wobei die Partikel mit einer geringeren Dichte eine höhere Infektiosität aufweisen als jene mit höherer Dichte.

### Lebenszyklus des HCV

HCV zeigt eine hohe Speziesspezifität (Mensch und Schimpanse) und infiziert nahezu ausschließlich Hepatozyten. Die Bindung und Internalisierung der Viruspartikel involvieren eine Vielzahl spezifischer Rezeptoren und Co-Rezeptoren (Abb. 3). Hierzu gehören der „low density lipoprotein receptor“ (LDLR), die Heparansulfat-Proteoglykane (HSPG), der „scavenger receptor class B type 1“ (SR-B1), das Tetraspanin CD81, Claudin 1 (CLDN1) und Occludin (OCLN), der Cholesteroltransporter *Niemann-Pick C1-like* (NPC1L1), die Rezeptortyrosinkinasen „epidermal growth factor receptor“ (EGFR) und „ephidrin type A receptor 2“ (EPHA2) sowie der Transferrinrezeptor (TfR; eine aktuelle Übersicht findet sich in [49, 50]). Nach clathrin-abhängiger Endozytose wird das RNA-Genom pH-abhängig in das Zytoplasma der Zelle freigesetzt, wo es als mRNA-Template für die Synthese des HCV-Polyproteins dient. Nach proteolytischer Prozessierung des Polyproteins erfolgt die Bildung der Replikonkomplexe (RCs) am sogenannten Membranous Web (MW). Das MW, ein charakteristisches Merkmal aller Flaviviren, besteht aus einem Netz von ER-Membranen, „lipid droplets“ (LDs) und Doppelmembranvesikeln (DMV). Letztere besitzen einen hohen Gehalt an Cholesterol und sind mit den viralen NS-Proteinen NS3, NS4B, NS5A und der viralen RNA assoziiert. Zudem enthalten sie den autophagosomalen Marker LC3, was auf eine Beteiligung des autophagosomalen Kompartiments an der Virusreplikation und auf Morphogenese hindeutet [51]. Die Replikation erfolgt durch die RNA-abhängige RNA-Polymerase NS5B sowie unter Beteiligung weiterer zellulärer Faktoren über ein (−)-Strang-Intermediat. Die Assemblierung der Virionen findet an der Oberfläche der LDs statt. Hierfür wird die genomische RNA zur Oberfläche der LDs transportiert [52], wo sich die virale RNA mit Core-Proteinen zu Nukleokapsiden assembliert. Diese werden unter Beteiligung von E1 und E2 in das ER abgeschnürt und verlassen schließlich die Zelle. Ob die Freisetzung über den klassischen sekretorischen Weg erfolgt oder über den ESCRT-abhängigen Weg unter Beteiligung des autophagosomalen Kompartiments, bedarf weiterer Klärung [53, 54].

### Pathogenese einer viralen Hepatitis C

HCV stellt, zusammen mit HBV, weltweit eine der Hauptursachen für akute und chronische Lebererkrankungen dar und zählt zu einer der häufigsten krebsassoziierten Todesursachen. Die akute Infektion verläuft meist asymptomatisch oder mit grippeähnlichen Symptomen, die bei etwa 15 % der Erkrankten von selbst ausheilt. In 60–85 % der HCV-infizierten Personen kommt es zur Ausbildung einer chronischen Hepatitis C. Aufgrund der HCV-induzierten Immunpathogenese, die mit einer persistenten Inflammation des Lebergewebes und einem erhöhten ROS-Siegel einhergeht, kommt es zu einer Verminderung der Leberregeneration, was unbehandelt in der Ausbildung einer Leberfibrose/-zirrhose endet [41]. Bei etwa 15–20 % der chronisch Erkrankten entwickelt sich im Laufe der Infektion nach 20–30 Jahren eine Leberzirrhose. Ausschlaggebend für die Progression der Leberzirrhose sind Wirts- und Umweltfaktoren, wie z. B. das Alter der Infektion, das Geschlecht, genetische Faktoren, Alkoholkonsum, Fettleibigkeit und Insulinresistenz. Bei 2–4 % der Patienten mit einer Leberzirrhose besteht das Risiko, an einem HCC zu erkranken (HCC-Rate pro Jahr; [44]). Eine detaillierte Darstellung der HCV-assoziierten Pathogenese erfolgt im Beitrag von Glitscher et al. in diesem Themenheft.

## Fazit

Weltweit leiden etwa 325 Mio. Menschen an einer chronischen Hepatitis. Die verantwortlichen Erreger haben gemein, dass sie spezifisch die Leber von Infizierten befallen, jedoch nicht eigenständig zu einem zytopathischen Effekt führen, sondern die Pathogenese auf der Entzündungsreaktion beruht. Neben akuten Verläufen kommt es nicht selten zu einer Chronifizierung der viralen Infektion mit den damit verbundenen Folgeerkrankungen. Bei genauer Betrachtung wird jedoch deutlich, dass es sich bei den verschiedenen Erregern einer viralen Hepatitis um grundlegend verschiedene Pathogene handelt. Somit Bedarf es im Umgang mit diesen Erregern einer differenzierten Betrachtung im Hinblick auf Epidemiologie, Nachweismethoden, Prävention und Therapie. So muss, trotz der Entwicklung robuster Therapien und der Verfügbarkeit von Vakzinen im Falle einzelner Erreger, die Forschung auf diesem Gebiet erheblich vorangetrieben werden. Dies gilt insbesondere auch für die Erreger, die armutsassoziiert auftreten. Die durch sie hervorgerufenen Erkrankungen müssen als bisher „vernachlässigt“ („neglected diseases“) angesehen werden.
